# The protective autophagy activated by GANT-61 in MYCN amplified neuroblastoma cells is mediated by PERK

**DOI:** 10.18632/oncotarget.24214

**Published:** 2018-01-13

**Authors:** Jing Wang, Siqi Huang, Ruicheng Tian, Jing Chen, Hongxiang Gao, Chenjie Xie, Yuhua Shan, Zhen Zhang, Song Gu, Min Xu

**Affiliations:** ^1^ Department of Surgery, Shanghai Children's Medical Center, School of Medicine, Shanghai Jiaotong University, Shanghai, 200127, China; ^2^ Pediatric Translational Medicine Institute, Shanghai Children's Medical Center, School of Medicine, Shanghai Jiaotong University, Shanghai, 200127, China; ^3^ Shanghai Pediatric Congenital Heart Disease Institute, Shanghai Children's Medical Center, School of Medicine, Shanghai Jiaotong University, Shanghai, 200127, China

**Keywords:** neuroblastoma, autophagy, PERK, MYCN-amplified, GANT-61

## Abstract

The proto-oncogene MYC can trigger the unfolded protein response (UPR). The double-stranded RNA-activated protein kinase-like endoplasmic reticulum kinase (PERK), one of three primary branches of the UPR, is a key regulator of autophagy, promoting tumorigenesis. Upon activation of PERK, there is an increase in phosphorylation of the eukaryotic initiation factor-2 alpha (eIF2α), which in turn, activates the transcription factor-4 (ATF4), responsible for an increased expression of LC3, a common autophagy marker. PERK is repressed upon GLI1 and GLI2 induction. GANT-61 is an inhibitor of GLI1 and GLI2, known to reduce autophagy in MYCN non-amplified, but not in MYCN amplified neuroblastoma (NB) cells. In our study, we tested the effect of the joint administration of a PERK inhibitor (GSK2606414) and the GLI inhibitor GANT-61 to MYCN amplified and MYCN non-amplified NB cells. Our results suggest that inhibition of PERK impairs GANT-61 induced autophagy in NB cells with MYCN amplification, but had no effect on the MYCN non-amplified NB cells. In summary, PERK seems to be a good therapeutic target for NB. Inhibition of PERK reduces autophagy in MYCN amplified NB cells, thus amplifying the efficacy of the GLI inhibitor GANT-61 in reducing proliferation of this type of cancer cells.

## INTRODUCTION

Neuroblastoma (NB) is the most common extracranial solid tumor in early childhood with an estimated incidence of 1 per 8,000 to 10,000 births [1, 2]. According to the International Neuroblastoma Risk Group (INRG) classification system, NB prognosis varies enormously, depending on the patient's genetic background, with NB outcomes ranging from spontaneous regression cure to untreatable [3]. Therefore, the molecular mechanisms underlying NB require further investigation and development of novel treatment strategies. Among the genetic alterations associated with the development of NB, the most predominant is the amplification of the MYCN oncogene, which is linked to advanced cancer stages, unfavorable biologic features, and a poor prognosis [4, 5].

Other than genetic background, the aberrant activation of signaling pathways is also common during tumor development. The hedgehog (Hh) signaling pathway is important during early embryogenesis where it promotes cell growth and differentiation, tissue patterning, and vascularization [6, 7]. Aberrant activation of components of the Hh signaling pathway may promote the differentiation of several tumors and have been linked to cancer-related hallmark processes. Components of the Hh signaling pathway include the three ligand variates: sonic (SHH), Indian (IHH), and desert (DHH); the receptor-patched homolog 1 (PTCH1); the receptor smoothened (SMO); and the transcription factors of the GLI family zinc fingers (GLI1, 2, 3) [8, 9]. In NB, the overexpression of Hh signaling induces maturation of cancer cells [10, 11]. Due to its important role in tumorgenesis, anticancer drugs based on Hh ligand antagonists (e.g., 5E1 and robotnikinin), Smo inhibitors (e.g., GDC-0449 and IPI-926), and GLI transcriptional activity inhibitors (e.g., GANT-58 and GANT-61) have been developed [12–14].

In our previous work, we applied the GLI transcriptional activity inhibitor GANT-61 to MYCN amplified and not-amplified MYCN NB cells [15]. Our data suggested that GANT-61 is an effective inhibitor of NB cell proliferation. However, MYCN-amplified NB cells were less sensitive to the GANT-61 treatment, which might be due to enhanced autophagy in NB cells with MYCN amplification, which may induce cell chemotherapeutic resistance activity. Therefore, it is important to investigate the regulatory mechanism underlying autophagy in NB cells with MYCN amplification as a way to improve current therapeutic strategies.

Autophagy is a highly conserved process which degrades and recycles proteins and organelles to generate nutrition for cell metabolism and allows survival under unfavorable conditions [16]. However, enhanced autophagy may bring death to the cell, even to tumor cells [17]. This dual outcome of autophagy creates difficulties when predicting its effect on specific tumor cells at a specific stage. The understanding of autophagy is further complicated by the thousands of proteins and protein interactions that take part in the modulation of autophagy [18].

Previous reports illustrated that the Hedgehog signaling pathway could regulate autophagy in a protein kinase R-like endoplasmic reticulum kinase (PERK)-dependent manner, as PERK is repressed upon induction of GLI1 and GLI2 [19]. PERK (double-stranded RNA-activated protein kinase-like ER kinase) is one of the three mechanistically distinct branches of the unfolded protein response (UPR) together with the activation of the transcription factor 6 (ATF6) and the inositol-requiring enzyme (IRE1) [20]. The purpose of UPR is to balance the degradation of properly and improperly folded proteins in the lumen of the endoplasmic reticulum (ER). Once UPR stress is excessively prolonged, UPR induces cell death via the three transducers described above [21] either by autophagy or apoptosis, in which PERK has been extensively involved [22, 23]. UPR activation has been associated with the development of many cancer types, including lung, liver, and breast [24]. The deletion of the UPR component PERK was reported to delay *Neu*-dependent mammary tumor development and reduce lung metastases [25]. Conversely, PERK activation promotes MYC-induced cell transformation through autophagy [26]. Given its role in the modulation of tumorgenesis and drug resistance, PERK may become a new target for cancer therapy.

However, the inhibition of GLI transcriptions might activate PERK-dependent autophagy in NB cells with MYCN amplification. In this study, we investigate the effect of the joint application of GANT61 and a PERK inhibitor or a PERK siRNA (reducing PERK protein expression) on MYCN-amplified NB cells and discuss the potential of this strategy in therapeutics.

## RESULTS

### PERK inhibitor treatment reduced PERK activity in NB cells

Several lines of evidence suggest a pivotal role of PERK in tumor progression [27]. A range of cellular stress signals may trigger the disruption of ER homeostasis and activate the Unfolded Protein Response (UPR), ultimately leading to tumor development. The activated protein kinase RNA-like endoplasmic reticulum kinase (PERK), a main branch of the UPR, leads to the phosphorylation of the Eukaryotic Initiation Factor 2 alpha (eIF2α). Subsequently, eIF2α activates ATF4, directly involved in the modulation of autophagy.

As previous studies reported that PERK is repressed upon Gli1 and Gli2 induction [28] and that GANT-61 is an inhibitor of Gli1 and Gli2, we hypothesized that GANT-61 activates PERK through the induction of the PERK-eIF2α-ATF4 pathway.

**Figure 1:**
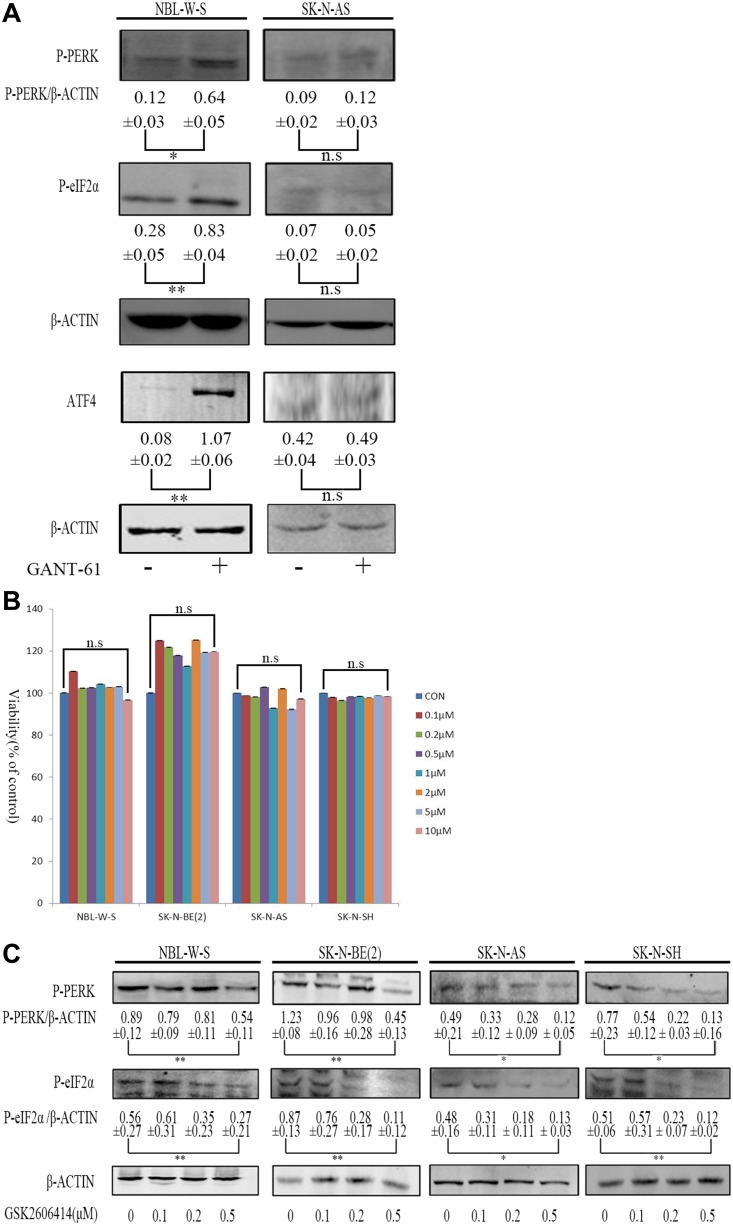
Assessment of GSK2606414 cytotoxicity and inhibitory on NB cell lines (**A**) NBL-W-S and SK-N-AS NB cells were treated with 10μM GANT-61 during 48 h. Western blots evaluated protein expression of PERK, P-eIF2α, ATF4. The PERK/β-ACTIN, P-eIF2α/β-ACTIN, and the ATF4/β-ACTIN ratio. The results are presented as mean ± SD of three independent experiments. ^*^P < 0.05, ^**^P < 0.01, n.s; is no statistical significance. (**B**) NBL-W-S and SK-N-BE(2) SK-N-AS and SK-N-SH NB cells were treated with 0.1-10 μM GSK2606414 for 3 h. Cytotoxicity of GSK26064141 was measured using the CCK8 assay. The percentage of viability cells was calculated as a ratio between treated and control cells. The results are presented as mean ± SD of three independent experiments. n.s, no statistical significance; CON control. (**C**) NBL-W-S, SK-N-BE(2), SK-N-AS and SK-N-SH NB cell lines were treated with different concentrations of GSK2606414 for 3h. P-PERK, P-eIF2α protein levels measured by Western blot. The inhibitory effects of GSK2606414 on PERK and eIF2α activity are presented as mean ± SD of three independent experiments. ^*^P < 0.05, ^**^P < 0.01.

We treated NBL-W-S NB cell lines (MYCN amplified) and SK-N-AS NB cell lines (MYCN non-amplified) with GANT 61 to evaluate changes in PERK- eIF2α-ATF4 pathway induction levels. Figure 1A shows that on the NBL-W-S NB cell lines (MYCN amplified) GANT-61 was able to induce the PERK-eIF2α-ATF4 pathway, but not on the SK-N-AS NB cell lines (MYCN non-amplified). GSK2606414 was reported as a specific inhibitor of PERK. To examine the effect of GSK2606414 in NB cells, we added GSK2606414 to NBL-W-S and SK-N-BE(2) NB cell lines (MYCN amplified) and to SK-N-AS and SK-N-SH NB cell lines (MYCN non-amplified) at various concentrations (0.1–10 μM). As GSK2606414 added at higher concentrations did not affect NB cells proliferation (Figure 1B) we repeated the assay using lower concentrations (0.1–0.5 μM) of GSK2606414 to NB cells. Lower concentrations of GSK2606414 significantly inhibited the levels of activation of PERK as it can be inferred by the reduction of P-PERK, P-eIF2α protein expression levels (Figure 1C).

### PERK inhibitor may block GANT-61-induced cell autophagy in MYCN-amplified NB cells

**Figure 2:**
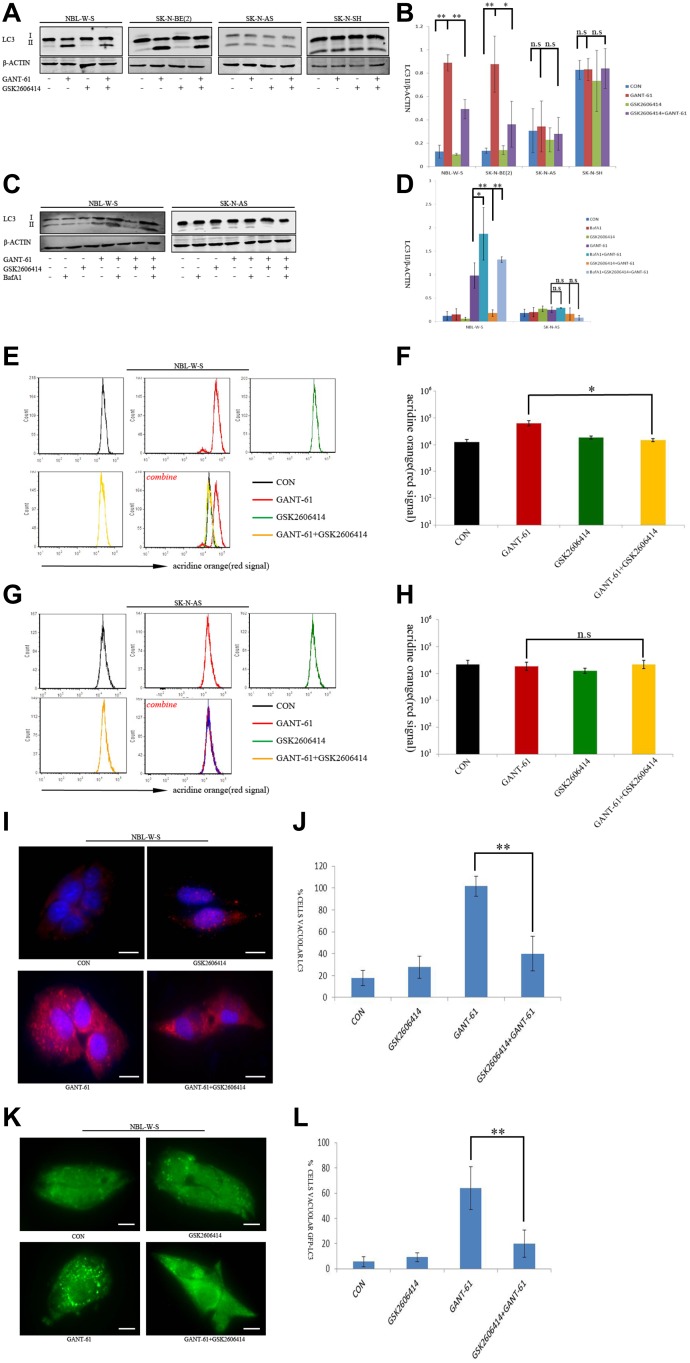
GSK2606414 inhibits GANT-61 induced cell autophagy in MYCN amplified NB cells (**A**) Assessment of LC3 conversion by LC3 immunoblotting. Membranes were reprobed with β-actin antibody. Four cell treatments CON (non-treatment), GANT-61 (10 μM GANT-61 treatment 48 h), GSK2606414(0.5 μM GSK2606414 treatment 3 h), GSK2606414+GANT-61 (0.5 μM GSK2606414 pretreatment 3 h with 10 μM GANT-61 treatment 48 h) were tested in NBL-W-S and SK-N-AS cells (**B**) The LC3-II/β-ACTIN ratio was plotted as histogram (mean ± SD), ^*^P < 0.05, ^**^P < 0.01. (**C**) Effect of lysosomal inhibitor BafA1 on autophagic flux induced by treatment with GANT-61 alone and by pre-treatment GSK2606414 with GANT-61. LC3 immunoblotting to evaluate LC3 conversion. NBL-W-S and SK-N-AS cells were first treated with 200nM BafA1 for 30 min and then treated with 0.5 μM GSK2606414 for 3 h followed by treatment with 10μM GANT-61 for 48 h. (**D**) The LC3 II/β-ACTIN ratio of Figure 2C was plotted as a histogram (mean ± SD), ^*^P < 0.05, ^**^P < 0.01, n.s., no statistical significance. (**E**) Flow cytometry analysis of AO stained NBL-W-S cells. NBL-W-S cells treated with indicated drugs. CON, control. (**F**) Flow cytometry histogram of AO stained NBL-W-S cells treated with the indicated drug. Data are expressed as the mean ± SD of three independent experiments. ^*^P < 0.05. CON, control. (**G**) Flow cytometry analysis of AO stained SK-N-AS cells. NBL-W-S cells treated in different drug treatment situations. CON, control. (**H**) Flow cytometry histogram of AO stained SK-N-AS cells treated with the indicated drug. Data are expressed as the mean ± SD of three independent experiments. n.s, no statistical significance. CON, control. (**I**) Immunofluorescence with the LC3 antibody on NBL-W-S cells after indicated drugs treatment. Scale bar, 20 μm. CON, control. (**J**) Quantification of cells with a number of LC3 aggregates five times higher than the basal level in the microscopy figure, ^**^P < 0.01. CON, control. (**K**) NBL-W-S cells were transfected with GFP-LC3 plasmids, the transfected cells were treated with different drugs. Accumulation of GFP-LC3 aggregates was analyzed by microscopy. CON, control. (**L**) Quantification of cells with GFP-LC3 aggregates, bars indicate the standard deviation of at least three measurements. ^**^P < 0.01. Scale bar 20 μm. CON, control.

There are two forms of the Light Chain 3 (LC3) proteins in various cells: LC3-I and LC3-II. The conversion of the soluble form of LC3-I to the autophagic vesicle-associated form LC3-II is a commonly used marker for auto-phagosome formation. We found a significantly increased LC3-II level after GANT-61 treatment in MYCN amplified NB cells NBL-W-S and SK-N-BE(2) [29]. However, the GSK2606414 treatment had no significant effect on the LC3-II level (Figure 2A). Importantly, GANT-61-induced increase in LC3-II levels was significantly blocked by GSK2606414 in MYCN amplified NB cells (Figure 2A). Moreover, the addition of GANT-61 or GSK2606414 had no effect on the levels of cleavage of LC3-II in MYCN non-amplified NB cells (Figure 2A, 2B). These results suggest that the joint effect of GANT-61 and GSK2606414 on the regulation of autophagy is MYCN-dependent.

Autophagy is a dynamic, multistep process, encompassing autophagosome formation, autolysosome formation, and autophagic substrate degradation, sometimes referred to as autophagy flux. Thus, we applied a lysosomal proton pump inhibitor BafilomycinA1 (BafA1) to assess the dynamics of the autophagy flux in NB cells. Dynamic change of autophagy flux was monitored in both NBL-W-S and SK-N-AS cells. As shown in Figure 2C, a higher level of LC3-II was detected in NB cells with both GANT-61 and BafA1 treatment, than in NB cells treated with GANT-61 alone (NBL-W-S), or in cells treated with a joint treatment of GSK2606414 with GANT-61. However, no significant variation of LC3-II expression was detected in SK-N-AS cells. The densitometry ratios of LC3II/β-ACTIN (Figure 2D) suggest that the autophagy flux was activated. The formation of acid vesicular organelles (AVOs) is a characteristic feature of autophagic cells. In acid compartments, such as lysosome and autolysosomes, the fluorescence of AO switches from green to red color. We performed flow cytometric analysis after AO staining. The red fluorescence signal after GANT-61 treatment of was stronger than that obtained after treatment with GSK2606414 alone or that obtained after the joint treatment of GANT-61 with GSK2606414 (Figure 2E) in NBL-W-S cells. Figure 2E is a histogram representation of Figure 2F. However, no significant fluorescence change was observed in SK-N-AS after any of the treatments (Figure 2G, 2H). The data suggest that GANT-61 significantly activated autophagy reflux, in a PERK-depended way in MYCN-amplified NB cells, but not in MYCN non-amplified NB cells.

The autophagosomal formation was also examined by immunofluorescence in NBL-W-S cell lines. GANT-61 significantly increased endogenous LC3 aggregates in NBL-W-S cell lines. However, pretreatment with GSK2606414 followed by treatment with GANT-61 for 48 h resulted in decreased levels of endogenous LC3 aggregates compared to those obtained after treatment with GANT-61 alone (Figure 2I, 2J). In addition, as overexpressing GFP-LC3 patterns compared to does obtained with endogenous LC3, we transfected NBL-W-S cells with GFP-LC3 plasmid to monitor autophagic induction. After GANT-61 treatment, we observed a change in the distribution pattern in the cytoplasm from a homogeneous distribution to aggregates in a large number of cells. However, we observed that the number of GFP-LC3 aggregates significantly decreased in NBL-W-S NB cells after the joint treatment of GSK2606414 with GANT-61 (Figure 2K–2L). Our data suggest that a pretreatment of GSK2606414 inhibits GANT-61-induced autophagy in MYCN amplified NB cells.

### PERK inhibitor reduced GANT-61-induced autophagy to upregulate cell cytotoxicity and apoptosis in MYCN amplified NB cells

**Figure 3:**
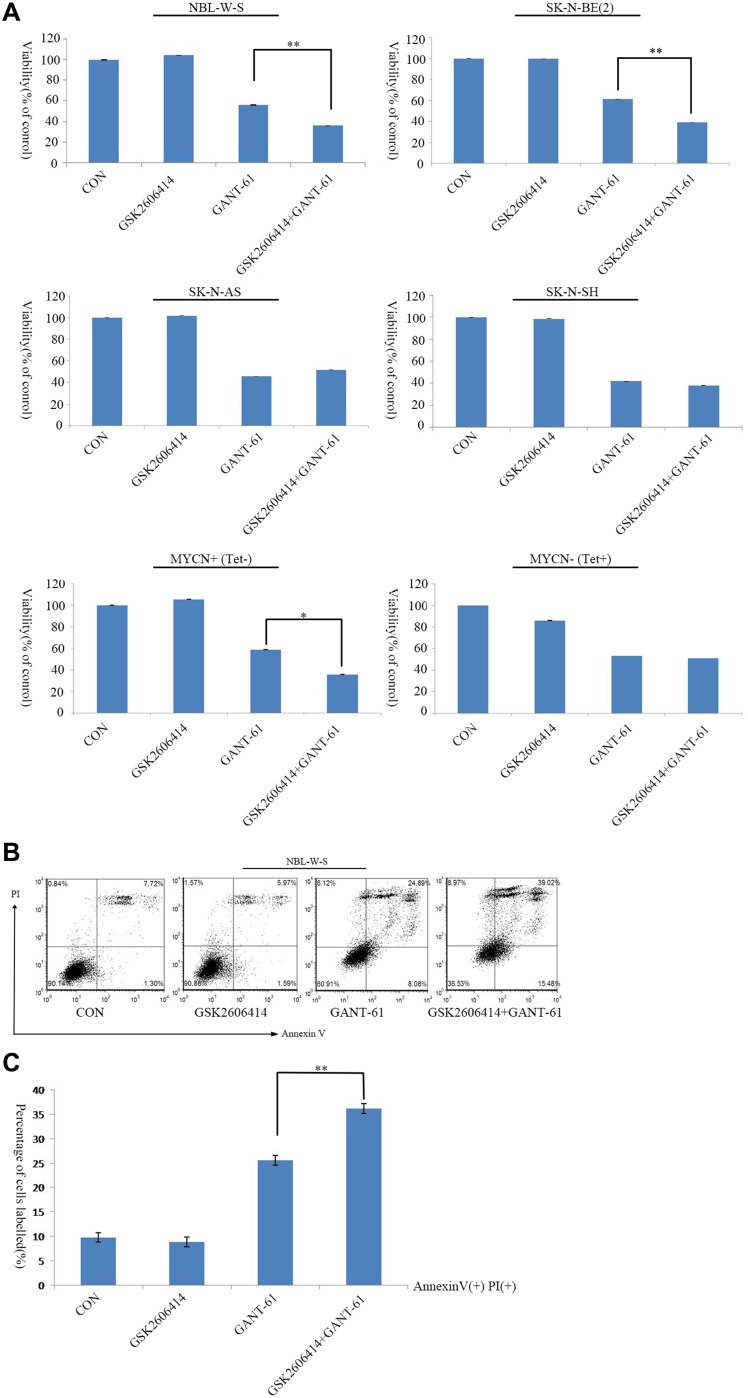
GSK2606414 enhanced GANT-61 cell cytotoxicity and apoptosis in MYCN amplified NB cells (**A**) NBL-W-S, SK-N-BE(2), SK-N-AS, SK-N-SH, MYCN+(Tet-) and MYCN-(Tet+) cell lines were treated with CON, 0.5 μM GSK2606414 3 h, 10 μM GANT-61 48 h, and pretreatment GSK2606414 3 h followed by GANT-61 treatment for 48 h. Cell viability was measured by a CCK8 assay. Data are expressed as the mean ± SD, ^*^P < 0.05, ^**^P < 0.01. CON, control. (**B**) Representative flow cytometry detection of apoptosis by concurrent staining with Annexin V-FITC and PI. NBL-W-S cells were treated as described previously. CON, control. (**C**) Histogram of flow cytometry results as obtained from three independent experiments. ^**^P < 0.01. CON, control.

GANT-61 was reported to have a cytotoxic effect on NB cell lines [30]. We found that pretreatment of GSK2606414 substantially affected GANT-61-induced cell cytotoxicity, and significantly reduced cell viability. Treatment with 10μM GANT-61 for 48 h resulted in 53.2% NBL-W-S live cells and 61.7% SK-N-BE (2) live cells. However, pretreatment of GSK2606414 before GANT-61addition resulted in 36% live NBL-W-S cells and 39% live SK-N-BE(2) cells. The counts in living cells after GSK2606414 pretreatment followed by GANT-61-are significantly lower than those resulting from GANT-61 treatment alone in MYCN amplified NB cells. Moreover, cytotoxicity in MYCN non-amplified NB cells was not affected by the joint treatment of GSK2606414 with GANT-61. Additionally, the Tet21N conditional MYCN expression cells were treated with the PERK inhibitor GSK2606414 and GANT-61 in the presence (MYCN-) or absence (MYCN+) of tetracycline. The cell viability assay showed that in MYCN(+) cells GSK2606414 could upregulate GANT-61-induced cell death. Conversely, GSK2606414 could not upregulate GANT-61 cytotoxicity in MYCN (−) (Figure 3A).

GANT-61-induced cell cytotoxicity is mediated through apoptosis. Therefore, we hypothesized that the PERK inhibitor (GSK2606414) also induces cell death through apoptosis. We used Annexin V-PI double staining to assess apoptosis. The number of apoptotic cells increased after pretreatment with GSK2606414.The sum of the early apoptotic cells (PI-/Annexin-FITC+) and the late apoptotic cells reached 54% (Figure 3B). The average of three independent experiments are shown in Figure 3C. The results suggest that GSK2606414 increased GANT-61-induced apoptosis.

### PERK knockdown expression inhibited GANT-61-induced autophagy in MYCN amplified NB cells

**Figure 4:**
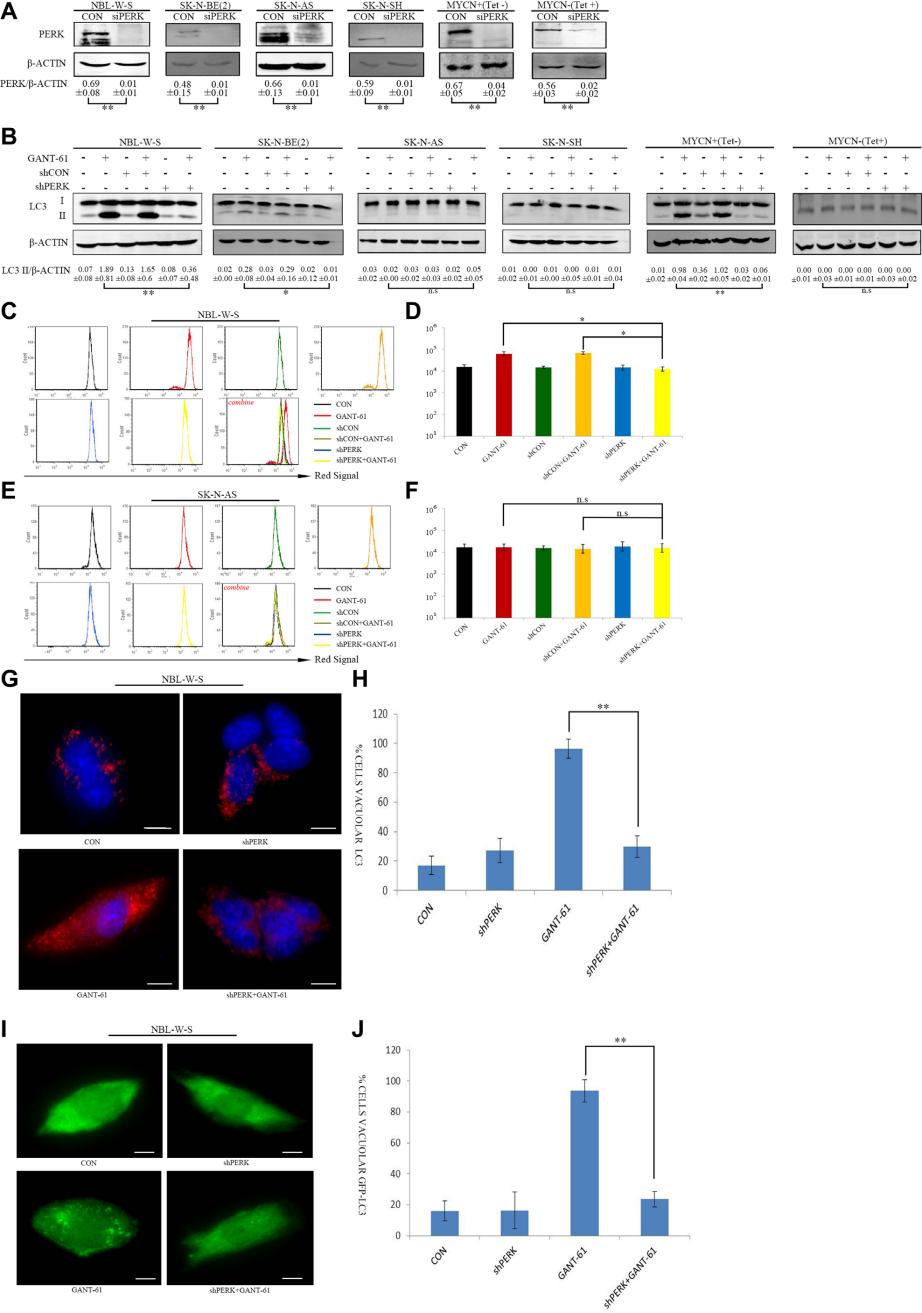
Knockdown PERK inhibits GANT-61 induced autophagy in MYCN amplified NB cells (**A**) PERK knockdown expression was verified by Western blot analysis with NBL-W-S, SK-N-BE (2), SK-N-AS, SK-N-SH and MYCN+(Tet-), MYCN-(Tet+) cells. Cells were transfected with shPERK plasmid, shPERK: PERK shRNA knockdown. Data are expressed as the mean ± SD, ^**^P < 0.01. (**B**) Knockdown PERK significantly decreased GANT-61 induced LC3 conversion, plasmids transfected cells were treated with 10 μM GANT-61 48 h, the LC3 II/β-ACTIN ratio was listed under blots. Data are expressed as the mean ± SD of three independent experiments. ^*^P < 0.05, ^**^P < 0.01. (**C**) Flow cytometry detection of AO staining NBL-W-S cells treated with the indicated drug. (**D**) Flow cytometry histogram of AO stained NBL-W-S cells treated with the indicated drugs. Data are expressed as the mean ± SD of three independent experiments. ^*^P < 0.05. CON, control. (**E**) AO stained SK-N-AS cells were analyzed by flow cytometry. SK-N-AS cells were treated with the indicated drugs. (**F**) Flow cytometry analysis of AO stained SK-N-AS cells treated with the indicated drug was plotted as an histogram (mean ± SD). n.s, no statistical significance. (**G**) Immunofluorescence for LC3, LC3 staining (red) in cytosol and nucleus is represented by blue (DAPI) staining. Scale bar 20μm. (**H**) Quantification of LC3 immunofluorescence expression in NBL-W-S. Immunofluorescence of LC3 staining results are presented as mean ± SD, ^**^P < 0.01, *N* = 3. (**I**) NBL-W-S transfected with the GFP fluorescent tagged LC3 plasmid, samples were examined by microscopy. Scale bar 20 μm. (**J**) Quantification of cells with GFP-LC3 puncta as histogram. Data are expressed as the mean ± SD of three independent experiments. ^**^P < 0.01.

Our results strongly suggest that GSK2606414 (PERK inhibitor) decreases GANT-61-induced autophagy in MYCN amplified NB cells. We subsequently confirmed this finding through the use of PERK knockdown cells with reduced PERK expression. First of all, we transfected lentiviral PERK shRNA plasmids to NBL-W-S and SK-N-BE(2) (MYCN-amplified NB cells) and in SK-N-AS and SK-N-SH NB (MYCN non-amplified cells). To further investigate the interaction between PERK and MYCN, we used the MYCN + (Tet-) and MYCN - (Tet+) cell lines (Figure 4A). We then assessed LC3 protein cleavage in NBL-W-S, SK-N-BE(2), SK-N-AS, SK-N-SH, MYCN+(Tet-), MYCN-(Tet+) NB cells after treatment with GANT-61 and shPERK. The results showed that the treatment with GANT-61 reduced the levels of LC3 conversion in NB cells with reduced expression of PERK. This reduction in LC3 conversion was greater than the one observed in MYCN-amplified NB cells (NBL-W-S, SK-N-BE(2) and MYCN overexpression NB cells MYCN+(Tet-)) (Figure 4B). However, PERK knockdown cells treated with GANT-61 had no significant effect on the levels of LC3 protein cleavage in MYCN non-amplified NB cells (SK-N-AS, SK-N-SH and MYCN-(Tet+) cells (Figure 4B). AO is widely used for detection of AVOs, an indicator of autophagy. Similarly to our Western blot results, we observed that there was an increase in the red fluorescent signal after treatment with GANT-61 in NBL-W-S. However, treatment with GANT-61 did not increase the red fluorescent signal in the PERK knockdown cells (Figure 4C, 4D). In SK-N-AS NB cells there was also no change in the intensity of the red fluorescent signal (Figure 4E, 4F). To confirm these data, we also examined the autophagosomal formation by immunofluorescence analysis in the MYCN amplified cells NBL-W-S. The data showed that GANT-61 significantly increased endogenous LC3 aggregates in the cells. However, in PERK knockdown NB cells, the treatment with GANT-61 induce a much lower level of endogenous LC3 aggregates (Figure 4G, 4H). In addition, we transfected NBL-W-S cells with GFP-LC3 plasmid to monitor autophagic induction. After GANT-61 treatment, we observed a distribution pattern change from an homogeneous distribution to the formation of aggregates in the cytoplasm in a significant number of cells (Figure 4I, 4J). However, in the PERK knockdown NBL-W-S cells, the treatment with GANT-61 lead to a distribution pattern change similar to that in the control group. Overall, these results support the theory that PERK knockdown cells had a lower level of autophagy expression after GANT-61 treatment in MYCN amplified NB cells.

### Knockdown of PERK reduced GANT-61-induced autophagy to upregulate cell cytotoxicity and apoptosis in MYCN amplified NB cells

**Figure 5:**
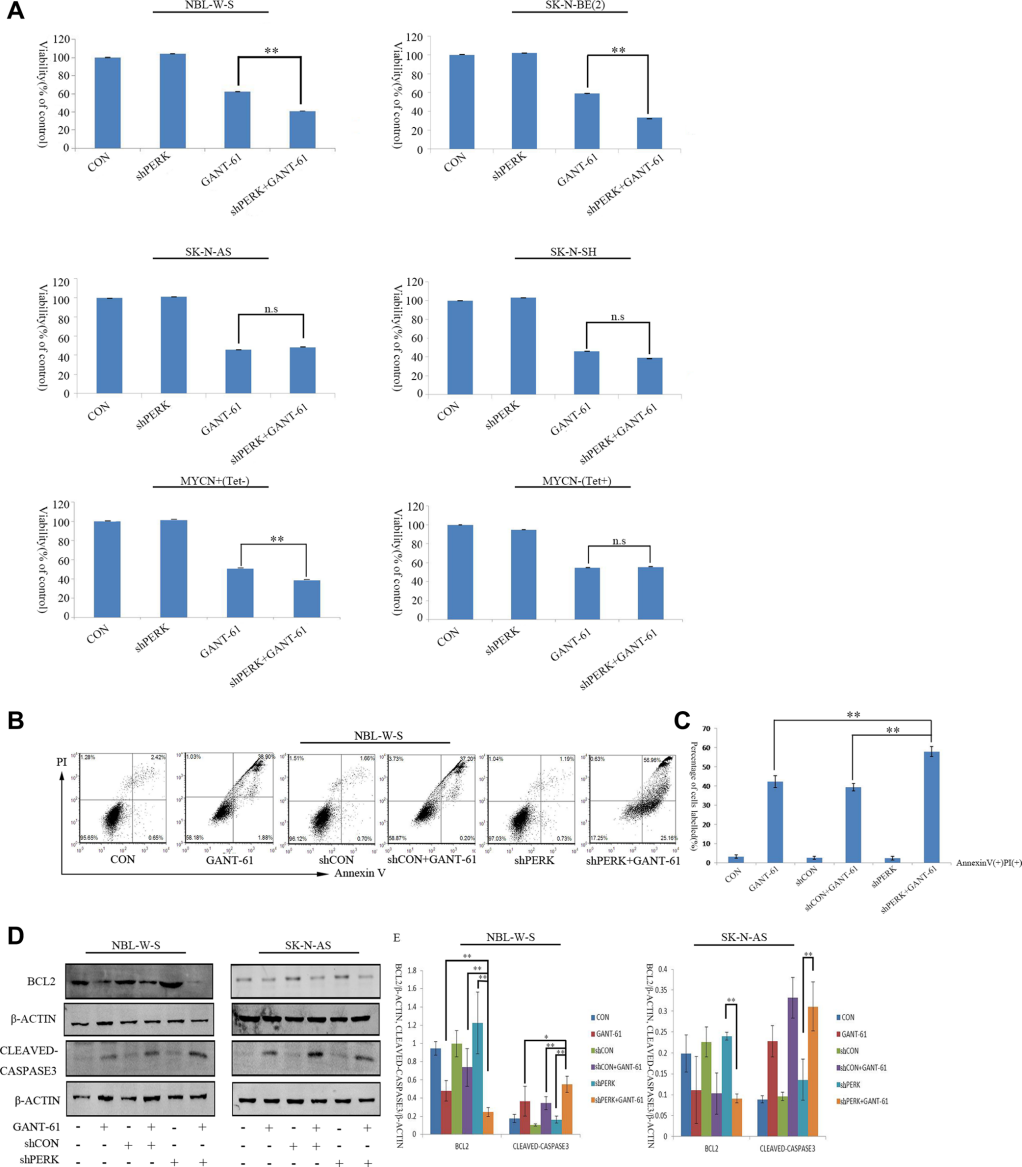
Knockdown PERK increases GANT-61 induced cell cytotoxicity and apoptosis in MYCN amplified NB cells (**A**) NBL-W-S, SK-N-BE(2), SK-N-AS, SK-N-SH, MYCN+(Tet-), MYCN-(Tet+), and PERK knockdown cells were treated with 10μM GANT-61 48 h, cell viability was measured by CCK8 assay. Data are expressed as the mean ± SD of three independent experiments. ^**^P < 0.01. (**B**) Representative flow cytometry detection of apoptosis by concurrent staining with Annexin V-FITC and PI. NBL-W-S cells were treated with 10μM GANT-61 48h. CON, control. (**C**) Flow cytometry histogram of Annexin V-FITC/PI double stained PERK knockdown NBL-W-S cells treated with GANT-61. (**D**) Levels of apoptosis were detected by Western blot analysis with anti-BCL2 and anti-cleaved CASPASE3 antibodies. (**E**) The BCL2/β-ACTIN and cleaved CASPASE3/β-ACTIN ratios (mean ± SD), ^*^P < 0.05, ^**^P < 0.01.

Our results described above suggest that the PERK inhibitor reduced GANT-61-induced autophagy in MYCN amplified NBL-W-S NB cells. We next examined the function of PERK on cell proliferation and apoptosis in NB cells after GANT-61 treatment. First, the effect of GANT-61 and PERK siRNA on NB cells proliferation was determined by a CCK8 assay (Figure 5A). In summary, data showed that 10 μM GANT-61 significantly inhibited cell proliferation in NBL-W-S (38%) and SK-N-BE(2) (42%) NB cells. Moreover, GANT-61 decreased cell viability in the PERK knockdown NBL-W-S (41%) and SK-N-BE(2) (36%) NB cells. More than that, MYCN overexpression MYCN+(Tet-) NB cells and MYCN amplified NB cells showed a similar rate of cell death. GANT-61 induced a 50% increase in cell death. In knockdown PERK cells, the dead cells increased to 62% (Figure 5A). However, in the MYCN non-amplified NB cells SK-N-AS, SK-N-SH and in the presence of tetracycline MYCN-(Tet+) NB cells the PERK siRNA could not induce a higher level of cell death than that obtained by the GANT-61 treatment alone (Figure 5A). Therefore, as our data strongly suggest that GANT-61-induced cytotoxicity occurs through apoptosis, we proceeded by assessing apoptosis using a flow cytometer (Figure 5B). Figure 5C is the quantitative representation of Figure 5B. After treatment with GANT-61, the percentages of annexin V-positive PI-positive (annexin V^+^PI^+^, late apoptotic) in NBL-W-S cells increased about 38.9%. The percentage of double-positive (annexin V^+^PI^+^, late apoptotic) in PERK siRNA NBL-W-S cells increased up to 57%. We also measured the expression of critical regulators of the apoptotic process by Western blot. Treatment with GANT-61 decreased the expression of apoptotic regulator B cell lymphoma leukemia2 (BCL2) and increased the level of active CASPASE 3 in MYCN amplified NBL-W-S cells. GANT-61 inhibited the expression of the apoptotic regulator BCL2. Moreover, it increased the level of active CASPASE 3 in the PERK siRNA transfected NBL-W-S cells (Figure 5D–5E). These results suggest that the apoptotic pathway is itself a major regulator of GANT-61-induced apoptosis in the PERK siRNA transfected MYCN-amplified NB cells.

### PERK inhibitor improves the efficacy of the GANT-61 treatment in MYCN amplified NB cells *in vivo*

**Figure 6:**
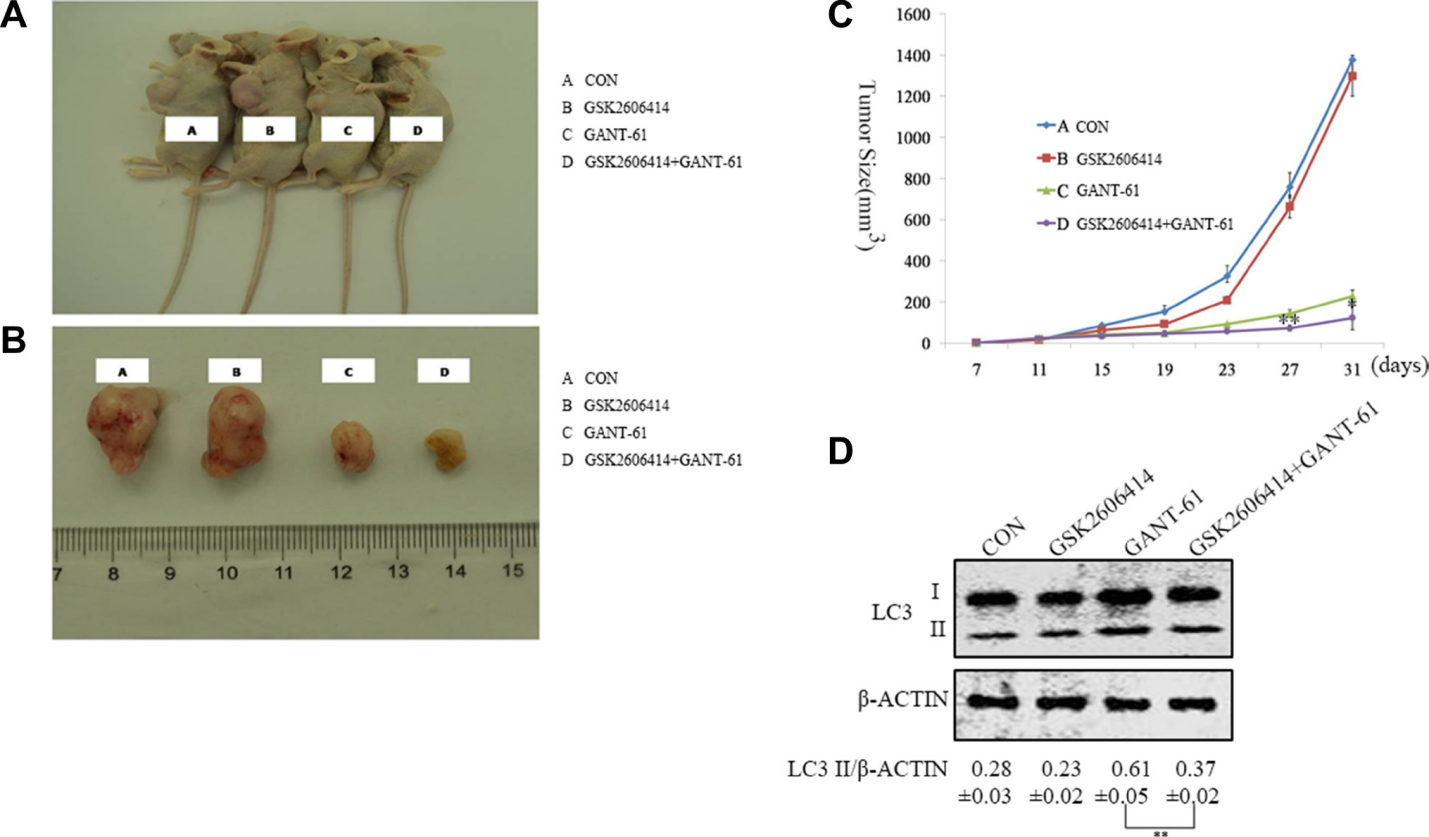
Inhibition of PERK enhances the efficacy of GANT-61 in MYCN-amplified NB cells *in vivo* (**A**) NBL-W-S cells were implanted into nude mice, drug treatment was assigned to mice 2 weeks later. Label A nude mice were treated with PBS as control; Label B nude mouse were treated with GSK2606414(30 mg/kg) twice daily, Label C nude mice were treated with GANT-61(50 mg/kg)three times per week, and Label D nude mice were treated with GSK2606414 and GANT-61.Tumor volume was measured every four days. (**B**) The mice were sacrificed after 31 days. Label A, B, C, and D nude mice were separately treated with PBS, GSK2606414 (30 mg/kg)., GANT-61(40 mg/kg), and GSK2606414 and GANT-61. (**C**) Approximate tumor volume in nude mouse was calculated using the following formula: length (L)×width (W)^2^×0.5. Data were represent mean ± SD. ^*, **^=significant difference between GSK2606414+GANT-61 group and the GANT-61 group,^*^P < 0.05, ^**^P < 0.01. (**D**) Western blot analysis. Effects of GANT-61 and GSK2606414 on the expression of LC3. Lysates were prepared from tumor tissues isolated from control, GSK2606414, GANT-61, GSK2606414 and GANT-61 treated mice. LC3 II/β-ACTIN ratios were listed under blots (mean ± SD), ^**^P < 0.01.

To determine the effects of PERK inhibitor GSK2606414 in the GANT-61 treated MYCN amplified NBL-W-S NB cells *in vivo*, mice received GSK2606414 and GANT-61 orally. Tumor growth was monitored, and after 31 days animals were sacrificed and tumors were harvested, measured and weighted. Tumor size in mice injected with GSK2606414 and GANT-61 was significantly smaller than those injected with GANT-61 alone (Figure 6A–6C). Additionally, we detected the expression of the LC3 protein in tumors by Western blot analysis. The results showed that GSK2606414 reduces LC3 II expression in the GANT-61 treated MYCN amplified NB cells (Figure 6D). These results suggest that the PERK inhibitor (GSK2606414) induced growth tumor in MYCN amplified NB treated with GANT-61 *in vivo* through inhibited autophagy.

## DISCUSSION

Neuroblastoma is a childhood malignant tumor of neural crest origin, arising in the sympathetic nervous system. Many results suggest that inhibitors of the Hh signaling pathway could suppress tumor growth through apoptotic induction [31–33]. Our previous findings confirmed that Hh signaling Gli1, Gli2 inhibitor GANT-61 not only induced apoptosis but also induced autophagy in NB cells. The level of the pro-survival autophagy is dependent on the MYCN expression level. The Hh signaling pathway is responsible for the correct development of the neural tubes and induction of ventral neural fate. In mammalian cells, three Gli orthologs, Gli1, Gli2, Gli3, mediate Hh transcriptional responses. PERK is repressed upon Gli1 and Gli2 induction, which was concomitant with a reduction in eIF2α phosphorylation at serine 51, both at basal conditions and following starvation, and consequently, PERK and EIF2α were implicated in the autophagy regulation [34]. Although the mechanism underlying EIF2α- mediated autophagy is largely unknown, it has been suggested that the EIF2α upregulation of ATG12 levels in the presence of expanded polyglutamines and ATF4 (transcription factor of EIF2α) is responsible for the increase of the transcription of ATG5 and LC3. Under this scenario, the PERK-EIF2α pathway is an important mediator in Hh-dependent autophagy.

In addition to PERK mediation of the Hh pathway, other regulatory mechanisms have to be considered. For example, some evidence suggested that in PERK-knockout MEFs, purmorphamine treatment still induced a degree of autophagy inhibition [35]. Moreover, some studies indicated that EIF2α phosphorylation is not the only signaling pathway involved in the control of autophagy by Hh signaling [36]. Indeed, in addition to PERK, there are three other kinases that phosphorylate EIF2α in mammalian cells, GCN2, PKR, and HRI. Overall, although numerous studies have focused on the molecular mechanism underlying PERK mediated autophagy, its specific role in different types of NB cells still required investigation.

MYCN oncogene, that shows amplification in many human cancers, has an important role as a regulator of critical cellular processes including proliferation, cell growth and differentiation [32, 34]. MYCN amplification is considered as an oncogenic marker for aggressive NB [33], and it is so far the most reliable prognostic factor for high-risk NB. Recent studies showed that cell-autonomous stress such as activation of the proto-oncogene MYC, can trigger the UPR and induce ER stress-mediated autophagy [35–37]. As one of three arms of the UPR response, the PERK/EIF2α-ATF4 promotes tumorigenesis by activating autophagy and enhancing tumor formation. PERK, an ER-resident protein that mediates signal transduction during ER stress, regulates the translation of the UPR by increasing phosphorylation of the EIF2α initiation factor subunit [24]. Therefore, the PERK-mediated UPR could be an attractive therapeutic target in MYC-driven cancers.

To understand the role of PERK in GANT-61-induced autophagy on MYCN-amplified NB cells, we used PERK inhibitor and PERK siRNA to treat MYCN-amplified NB cells. Our data show a reduction of GANT-61-induced autophagy when NB cells are pre-treated with a PERK inhibitor (GSK2606414) and in PERK knockdown cells (Figures 2, 4). Conversely, MYCN non-amplified NB cells were less susceptible to GANT61 than MYCN amplified NB cells. GANT-61 induced less autophagy in the MYCN non-amplified NB cells than in MYCN amplified NB cells (Figure 2A). Meanwhile our data also revealed that the PERK inhibitor (GSK2606414) or PERK siRNA cannot reduce GANT-61-induced autophagy in the MYCN non-amplified NB cells (Figures 2A, 4B), as the MYCN non-amplified NB cells cannot activate the downstream UPR response to the same extent as PERK. Therefore, the used of PERK inhibitor or of PERK siRNA had no effect on the levels of autophagy expression in MYCN-non-amplified NB cells. Throughout our research, we used SK-N-AS NB cells, the MYCN non-amplified NB cells (MYC expression). Although MYC could also mediate the activation of PERK, the functions of MYC and MYCN are different. Therefore, compared to MYC, MYCN shows a more targeted expression pattern, with temporal and tissue specificity. MYCN has a key role during neuroblastoma differentiation. Figures 4A, 4B, show SK-N-AS had high expression of PERK, but could not increase LC3 protein expression to the same extent as MYCN amplified NB cells, even though the LC3 level of SK-N-AS was higher than other MYCN non-amplified NB cells. This means that MYC overexpression, in the absence of MYCN, could induce PERK and LC3 activation but at much lower levels than those of MYCN amplified NB cells. We suggested that amplified MYCN is crucial for PERK–mediated autophagy. Meanwhile, inhibition of PERK increased cell death through apoptosis (Figure 5A, 5B). We found that the apoptotic pathway, including the expression of the genes BCL2 and CLEAVED-CASPASE3, was affected by PERK inhibition in the MYCN amplified NB cells. We speculate that inhibition of PERK could down-regulate the expression of BCL2 and CLEAVED-CASPASE3 protein levels in MYCN amplified NB cells. In MYCN non-amplified NB cells, the PERK inhibitor or shPERK did not affect the BCL2 or the CLEAVED-CASPASE3 protein levels after GANT-61 treatment (Figure 5D). This suggests that MYCN amplification and PERK inhibition greatly contribute to GANT-61-induced apoptosis.

Previous results showed that C-MYC and N-MYC overexpression are capable of inducing cytoprotective autophagy through the PERK-EIF2α-ATF4 arm of the UPR. Furthermore, GANT-61-induced autophagy depends on activation of the PERK gene. Our data revealed that the PERK inhibitor or PERK siRNA impaired GANT-61-induced pro-survival autophagy in MYCN amplified NB cells with, which greatly suggests that PERK could be a good therapeutic target for the therapy of NB patients with MYCN amplification.

## MATERIALS AND METHODS

### Cell culture

Four NB cell lines were used in this study. The NB cell lines NBL-W-S and SK-N-AS were obtained from the State Key Laboratory of Hematology Oncology of the Ministry of Health, Shanghai Children's Medical Center (China). The NB cell lines SK-N-BE (2) and SK-N-SH were purchased from the Shanghai Institute of Cell Biology, Chinese Academy of Sciences (China). The conditional MYCN-expression cell line SHEP Tet21N was used as a control, SHEP Tet21N cells were cultured for at least 48 hours in a tetracycline solution at a concentration of 1μg/ml (Sigma, UK) in order to switch off MYCN amplification. NBL-W-S, SK-N-BE(2), SK-N-SH, and SHEP Tet21N cells were maintained in DMEM (Dulbecco's Modified Eagle Medium) with high glucose (Life Technologies, USA). SK-N-AS cells were cultured in DMEM with low glucose (Life Technologies, USA). Both media were supplemented with 10% fetal bovine serum (FBS) (Life Technologies, USA) and 1% penicillin/streptomycin (Sigma-Aldrich Co, MO), and 200 μg/ml of G-418 antibiotic was added to the Tet21N and Tet21 media. Cells were cultured as a monolayer in a humidified atmosphere containing 5% CO_2_ at 37°C.

### Antibodies and reagents

β-ACTIN, CLEAVED-CASPASE3, PERK, phospho-eIF2α, ATF4 rabbit antibodies were purchased from Cell Signaling Technology, (USA). Phospho-PERK antibody was purchased from Abcam (USA), Light Chain 3B-LC3B rabbit antibody was purchased from Novus (USA). B cell lymphoma leukemia2-BCL2 rabbit antibody was purchased from Santa Cruz Biotechnology (USA). The secondary antibody, goat anti-rabbit IgG, was purchased from Rockland (USA). GANT61 were purchased from Sigma Chemical Co (USA), GSK2606414 were purchased from Sellckchem (USA).

### Cell proliferation assay

Cell proliferation was detected using the Cell Counting Kit-8 assay (CCK8, Dojindo Laboratories, Japan) according to the manufacturer's instructions. Briefly, cells were seeded in 96-well plates at the density of 10,000 cells per 100 μL of medium per well. After 48 h of treatment, CCK8 (10 μl/well) was added to each well and cells were incubated for 3 h at 37°C in the dark. After incubation, the absorption value of the individual wells was measured at 570 nm with a Glo Max-Multi Detection System (Promega, USA). The absorbance value (OD) of each well was converted to relative cell number using a standard curve.

### Western blot analysis

Total protein extracts were prepared in whole cell lysis buffer (50 mM HEPES, 150 mMNaCl, 1mM EGTA, 10 mM sodium pyrophosphate, 1.5 mM MgCl_2_, 100 mM sodium fluoride, 10% glycerol and 1% TritonX-100) containing a protein degradation inhibitor mixture (1 mM Phenylmethylsulfonyl fluoride, 10 μg/ml aprotinin, and 1 mM sodium orthocanadate). Protein concentrations were measured using the BCA protein assay kit (Bio-Rad, Hercules, CA, USA) according to manufacturer's instructions. A total of 40 μg of total protein was subjected to SDS-PAGE followed by a transfer onto PVDF membranes (Millipore, Bedford, USA). Membranes were blocked with blocking buffer for 1h and then incubated with various primary antibodies (1:1000) overnight at 4°C. After PBS washes, membranes were incubated with the IRDye 800-conjugated goat anti-rabbit IgG secondary antibody (1:2000) for half an hour at room temperature. Immunoreactive bands were visualized with a Licor Odyssey Infrared Imaging System (LI-COR Biosciences, USA).

### Annexin V binding assay

Flow cytometry (BD Biosciences, USA) was performed to detect cell apoptosis. The binding of annexin V-fluorescein isothiocyanate to externalized phosphatidylserine was used a measurement of apoptotic cells. The apoptosis detection kit (BD Biosciences, USA) based on Annexin-V -FITC/propidium iodide (PI) staining was used to detect apoptosis according to the manufacturer's instructions. Briefly, cells harvested by trypsinization were re-suspended with binding buffer at a density of 1 × 10^5^ cells per ml and incubated with 5 μL of FITC-conjugated Annexin-V and 5 μL of propidium iodide (PI). After intensive mixing, the tube was incubated for 15 min at room temperature in the dark. Finally, AnnexinV (+) PI (−) and Annexin V(+) PI(+) cells were detected under flow cytometry microscopy, and the total number of cells in each group were compared (as a percentage).

### Acridine orange staining assay

Acridine orange (AO) staining was used to visualize the volume of the cellular acidic compartments. AO is an acidotropic flourescent dye that stains DNA and cytoplasm components a bright green. Cells were treated with AO (1 μg/ml) in serum-free medium for 15 min at 37°C.

For AO staining analysis using flow cytometry, cells were washed and then illuminated with blue excitation light (488 nm). The mean fluorescence emission of green light (510–530 nm) and red light (650 nm) of 1 × 10^4^ cells were measured with a BD FACSCanto II Flow Cytometer (BD Biosciences, USA).

For AO staining analysis using fluorescence microscopy, an inverted fluorescence microscope was used (Leica DMI3000, Solms, Germany).

### Indirect Immunofluorescence

Cells, grown on coverslips, were fixed in a 3.7% paraformaldehyde PBS solution for 10 min at room temperature. Cells were then washed with PBS and blocked with 1% bovine serum albumin in PBS solution for 1 h. Subsequently, cells were incubated with the primary antibody, a rabbit polyclonal affinity purified anti-LC3 (dilution 1:400, Novus Biologicals, USA). Bound antibodies were detected by goat anti-rabbit IgG-H&L Cy3 secondary antibody (Abcam, Hongkong) for 1 h. Cells were examined by confocal fluorescence microscopy. Image analysis was done with Image-Pro Plus (Media Cybernetics, USA). The basal value of acid vesicular organelles (AVOs) is measured as the average number of fluorescent aggregates in untreated cells. Cells are considered actively autophagic when the AVO value is 5 times higher than the baseline value.

### Transfection of green fluorescent protein–light chain 3 plasmid

Cells were plated in 6-well plates and refreshed with serum- and antibiotic- free DMEM for 12 h before transfection. Cells were transfected with GFP-LC3 plasmid using Lipofectamine LTX reagent (Life Technologies, USA) according to manufacturer's instructions. Plasmid GFP-LC3 was a kind gift from Dr. Zhixueliu (Institute for Nutritional Sciences, SIBS, China). The cells were examined under a fluorescence microscope (Leica DM6000, Germany). Induction of autophagy was quantified by counting the percentage of cells in each group with a number of LC3 aggregates. GFP-LC3 aggregates were counted in three independent experiments, on over 30 cells.

### Virus packing and plasmid transfection

Lentiviral particles were produced by calcium phosphate transfection of lentiviral plasmids using the envelope vector pMD2.G and packaging vector pCMV-∆8.74 into 293FT cells (the molar ratio of plasmids is 2:3:3). Supernatant with viral particles was collected 48 hours after transfection. Lentiviral PERK shRNA vectors were purchased from Genechem (China). shRNA sequence listed below: PERK:5′-TTTGGAATCTGTCACTAAT-3′.

### Animal model of tumor xenografts

Nude mice (mice, 20–25 g) were used in this study. Mice were divided into four groups randomly (*n* = 5 per group). All cells were trypsinized and resuspended in 100 μl of PBS (containing 50 μl Matrigel). Tumor xenografts were generated by subcutaneous injection of 1 × 10^6^ cells into the flank of 6 weeks old BALB/c nu/nu mice. Mice were orally administered GANT-61 (50 mg/kg body weight), three times per week and GSK2606414 (30 mg/kg body weight) twice daily. Tumor dimensions were measured every 4 days, and after one month, all mice were sacrificed, and tumors were isolated for biochemical analysis.

### Statistical analyses

IC50 was calculated using Probit regression analysis. Each experiment was performed at least three times, and results were reported as mean ± standard deviation (SD). Results were statistically analyzed using ANOVA and Student's *t*-test. All statistical analyses were performed using the SPSS 17.0 software package (SPSS, Inc., Chicago, IL, USA). All *P*-values were two-sided, and *P* < 0.05 was considered to indicate a statistically significant difference.

## Abbreviations

AO: Acridine orange
AVOs: acid vesicular organelles
BafA1: Bafilomycin A1
Bcl-2: B cell lymphoma leukemia-2;CON
DMEM: Dulbecco's Modified Eagle Medium
DMSO: dimethylsulfoxide;FBS
FITC: fluorescein isothiocyanate
Hh: hedgehog;LC3
MDC: monodansylcadaverine
CCK8: cell counting kit-8
NB: neuroblastoma
NC: nitrocellulose
PI: propidium iodide
PVDF: polyvinylidene fluoride;PBS
SDS: sodium dodecyl sulfate
Sonic hedgehog (SHH): Indian hedgehog (IHH)
